# Disrupted Sensemaking—Understanding Family Experiences of Physical Restraints in ICU: A Phenomenological Approach in the Context of COVID-19

**DOI:** 10.3390/healthcare12121182

**Published:** 2024-06-12

**Authors:** Michele Flynch, Keville Frederickson

**Affiliations:** College of Health Professions, Lienhard School of Nursing, Pace University, Pleasantville, NY 10570, USA; tomassons@aol.com

**Keywords:** COVID-19, physical restraints, intensive care units, family experiences, psychological distress, patient-centered care

## Abstract

Background: The emergence of COVID-19 profoundly influenced the dynamics within intensive care units, significantly altering the patient–family experience. As the pandemic unfolded, the longstanding practice of using physical restraints for patient safety persisted, introducing new challenges in healthcare settings. This study explored the ramifications of these enduring safety measures on family members of ICU patients during the pandemic, illuminating their lived experiences and the psychological impact of seeing their loved ones restrained. Objectives: To explore family members’ lived experiences with physical restraints in the ICU during COVID-19 and inform improvements in patient-centered care. Methods: Utilizing hermeneutic phenomenology, the study engaged ten family members in detailed interviews to gain an understanding of their experiences with ICU physical restraints during COVID-19. Conducted at a northeastern U.S. hospital, the collected narratives underwent thematic analysis within a sensemaking framework, yielding a profound understanding of family perspectives. Results: Family members faced challenges in understanding and coping with physical restraints, revealing a need for improved healthcare system support for family sensemaking and well-being. Conclusions: The study advocates integrating empathetic communication and family engagement into ICU care practices, underlining the importance of sensemaking during healthcare crises.

## 1. Introduction

The emergence of COVID-19 as a global health crisis precipitated a series of rapid and profound transformations within the healthcare sector. These changes were most acutely felt within intensive care units (ICUs), which became focal points for the management of a disease with significant infectiousness and severity. Healthcare professionals worked tirelessly to save lives while contending with the evolving nature of the virus and its transmission. In this high-stakes environment, the longstanding practice of using physical restraints to prevent patients from compromising their treatment by pulling at medical devices gained renewed urgency and complexity as ICUs struggled to balance patient safety with emerging infection control protocols.

Historically, the application of physical restraints in critical care settings has been a delicate balance between ensuring patient safety and respecting the dignity and autonomy of the critically ill. Foundational studies, like those by Evans and Strumpf [1], have been instrumental in challenging the status quo, inspiring nursing reforms that aimed to minimize the use of restraints and prioritize more humane approaches to patient care. These important studies shed light on the moral and psychological impacts of restraint use, igniting a dialogue that has spurred ongoing reform.

The arrival of SARS-CoV-2, the virus causing COVID-19, required swift adjustments to patient management protocols, including those related to the use of physical restraints. A study by Crutchfield et al. revealed that prior to the pandemic, physical restraints were used in up to 75% of U.S. ICU patients to prevent treatment interference, despite known physical and psychological risks [2]. As ICUs adapted to the pandemic, the patient–caregiver dynamic underwent a fundamental shift. Infection control measures mandated a physical separation between patients and their families, a necessary move but one that introduced new challenges and exacerbated existing ones. Families, traditionally a constant in the caregiving milieu, were compelled to remain distant from their loved ones’ bedside, confronting profound implications.

Although the acute phase of the pandemic—characterized by high infection and mortality rates, intense media coverage, and pervasive fear—has somewhat abated, the ramifications of the pandemic’s impact on healthcare practices, particularly regarding the use of physical restraints, continue to be felt. It was vital to understand how contemporary healthcare measures, formulated in response to COVID-19, influenced the application of physical restraints. Additionally, it was important to examine the psychological impact of these measures on patients and, significantly, on their families.

Works conducted by Davidson, Jones, and Bienvenu [3] which focused on family responses to critical illness, highlighted the emotional and psychological toll that ICU stays can exact on family members. Similarly, the examination of Kross et al. [4] into depression and PTSD symptoms in family members post-ICU stay underscored the long-term impact of critical care stays and interventions on families. These studies pointed to an urgent need for a more comprehensive exploration of family well-being in ICU settings. Limited studies have explored family perspectives on ICU restraint use [5] Perez et al. underscores the need to understand their perceptions and psychological outcomes. This qualitative study initially aimed to explore patients’ lived experiences with restraints in the ICU. However, the pandemic necessitated a shift to focus on family members, as patient access became restricted. The study aimed to address the specific gap in understanding the experiences of families whose loved ones were physically restrained in ICU settings during the pandemic. It examined the coping mechanisms of and psychological impact on these families, both during the height of the crisis and its aftermath. The research question guiding this study became: What is the lived experience of family members who had a loved one physically restrained in the ICU during COVID-19?

The research employed a hermeneutic phenomenological approach, as detailed by van Manen [6], to interpret these experiences within the contexts of evolving healthcare protocols and patient-centered care. Furthermore, this study examined these experiences through the lens of Weick’s [7,8] sensemaking framework, which provided a valuable perspective of how families constructed and understood their experiences in complex and crisis-driven healthcare environments.

By broadening comprehension to encompass not only the direct effects on patients but also the consequential impacts on their families, this study makes a significant contribution to contemporary healthcare research. It illuminated how pandemic-induced changes in ICU practices, particularly those concerning physical restraints, have rippled through and affected family units. This research drew on insights from Abdeljawad and Mrayyan [9] regarding the contentious nature of physical restraints, as well as from Almeida et al. [10] concerning the application of nursing theories in the context of a pandemic. By integrating various strands of academic discourse, this research provides a narrative embedded in the extant healthcare crisis and offers valuable insights into current healthcare challenges and the ongoing debate over physical restraint use.

## 2. Methods

The methodology of this study was anchored in the hermeneutic phenomenological tradition, as established by van Manen [6]. This approach facilitated a profound exploration of the emotional and psychological experiences of family members whose loved ones underwent physical restraint in ICU settings during the COVID-19 pandemic. Employing this qualitative methodology allowed for an in-depth investigation into the complex realities of the participants’ lived experiences, yielding a detailed and interpretive analysis of the phenomena under study. The researchers engaged with the data through a rigorous iterative process, aimed at eliciting the essence of the participants’ experiences and understanding the meanings they ascribed to these difficult circumstances.

### 2.1. Participant Recruitment and Ethical Considerations

Participants were purposively recruited through the ICU units of a large hospital in New York City, a region heavily impacted by COVID-19. A total of fifteen potential participants, who were family members of ICU patients that experienced physical restraint during the pandemic surge, were approached. These individuals were identified through hospital medical records. Of the fifteen, ten agreed to participate. The five who declined cited reasons such as emotional distress, lack of time, or unwillingness to revisit the experience. The sample included family members of patients with various diagnoses, including COVID-19, to reflect the diverse range of ICU experiences during the pandemic.

Although the study was initiated before the pandemic, data collection took place from June to August 2020, once COVID-19 was underway, with interviews taking place within 2 weeks of the patients’ ICU admissions. Input was sought from family members due to the severity of the patients’ illnesses and the pandemic-related restrictions, which made direct interviews with patients impractical.

Ethical approval was secured from the Institutional Review Boards of both the hospital and the sponsoring university to ensure the study conformed with ethical standards and guidelines. Those who agreed to participate provided electronic informed consent, acknowledging their understanding of the study’s purpose and their right to withdraw at any time.

### 2.2. Data Collection Procedures

Data collection was meticulously carried out using a series of comprehensive, semi-structured interviews, which were facilitated via a secure, HIPAA-compliant video-conferencing service. This method was specifically chosen to comply with the social distancing measures during the pandemic. The initial probing question directed to participants was carefully crafted: “Tell me about a time when your family member was in the hospital ICU and was restrained”. Such an open-ended invitation encouraged participants to speak candidly and in detail about their experiences. In phenomenological studies, follow-up questions play a crucial role in deepening the understanding of the participants’ experiences. The research protocol included the development of tailored follow-up questions, which stemmed organically from each participant’s account. These subsequent questions were designed to explore specific aspects of the initial narrative more thoroughly. For instance, if a participant mentioned feeling helpless when they saw their loved one restrained, the follow-up questions might have included:

- “Can you describe what was happening at that moment?”
- “How did you react to seeing your family member restrained?”
- “What thoughts were going through your mind?”
- “How did the healthcare staff explain the use of restraints to you?”

These follow-up questions were crafted to probe more deeply into the participants’ emotional and cognitive responses, their interactions with healthcare providers, and the broader context of their experiences. This approach enriched the understanding of the individual’s experience without leading or restricting their response, ensuring that the data collected were both authentic and comprehensive. The flexibility of follow-up questions allowed the researchers to adapt to the flow of the conversation, making sure that all relevant aspects of the experience were explored in depth.

### 2.3. Participant Demographics

The study engaged a diverse sample of 10 family members, predominantly female (90%) and all over 21 years old, whose loved ones were physically restrained in the ICU during the COVID-19 pandemic. Participants were purposively selected to capture a range of patient diagnoses, including both COVID-19 and non-COVID-19 conditions, reflecting the spectrum of ICU experiences during this time.

The patients connected to these family members were aged 57–90 and primarily admitted for acute respiratory conditions. Six patients died during their ICU stay, while four were discharged to post-acute care facilities or home. The sample represented a rich cross-section of nationalities (see Table 1), providing cultural context to the findings.

Due to pandemic-related restrictions and the severity of patient illnesses, interviews were conducted with family members rather than patients directly. This approach offered a unique window into the ripple effects of restraint use on families during a profoundly challenging time.

Table 1 presents detailed demographic characteristics of the family member participants and their loved ones in the ICU, including age, diagnosis, unit location, and outcomes. The gravity of the medical situations faced, combined with the visitation restrictions, underscores the profound psychosocial impact these experiences had on the family members interviewed.

### 2.4. Data Analysis

The data were analyzed using NVivo 1.0 to facilitate systematic management and scrutiny of the qualitative data. The research adhered to van Manen’s [6] hermeneutic phenomenological methodology, involving a thorough line-by-line thematic analysis. This analysis entailed a deep engagement with the transcribed interviews to highlight and annotate critical statements and thematic elements that captured the essence of the participants’ experiences. These annotated segments were then compiled to form coherent themes that served as the basis for the study’s empirical findings. No new codes emerged after coding the transcript of P06’s interview. The interviews and early analysis of the data collected from the remaining participants, P07 to P10, confirmed that data saturation and redundancy had been reached because no new themes emerged.

To ensure the study’s trustworthiness and rigor, multiple methodological strategies were employed. Member checking was a pivotal part of this process, enabling participants to verify the accuracy of their interview transcripts and ensure the data’s fidelity to their lived experiences. The participants confirmed that the emergent themes were congruent with their personal narratives.

Further enhancing the study’s analytical rigor, an external auditor with expertise in phenomenological research was engaged to review a subset of the data and validate the identified themes. This external review functioned as a quality control measure, adding a layer of verification and bolstering the credibility of the thematic analysis.

## 3. Results

The analysis, conducted through a hermeneutic phenomenological framework, involved a thorough examination of the participants’ experiences. The process began with the identification of 16 initial themes that emerged from the data. These themes were then grouped into theme categories based on their shared characteristics and the overall essence of the participants’ narratives. From these theme categories, five essential themes were derived, representing the core aspects of the participants’ experiences. The theme categories served as a foundation for the development of the essential themes, which encapsulate the fundamental elements of the lived experiences of family members with loved ones who underwent physical restraint intervention in the ICU during the COVID-19 pandemic.

These essential themes were instrumental in elucidating the layered emotional and ethical quandaries that participants encountered. Each essential theme is detailed in Table 2, along with its corresponding theme category and general themes. The essential themes are further elaborated upon in the discussion section that follows. Extracts from participant testimonies serve to substantiate these themes, providing a resonant depiction of the diverse and complex experiences these family members underwent. These direct quotes offer an authentic narrative that reinforces the thematic findings, contributing to a comprehensive understanding of the participants’ multifarious challenges during a tumultuous time.

### Articulation of Essential Themes through Participant Voices

The study documented the lived experiences of family members whose loved ones were subjected to physical restraints in the ICU during the COVID-19 pandemic. Through hermeneutic phenomenological analysis, several essential themes were identified which captured the complex interplay of emotions, ethical considerations, and need for clear communication.

**Essential Theme 1: Managing Complex Hospital Care Challenges Throughout the Continuum of Hospital Care**

This theme encompassed the complex emotional burden faced by family members during the entire hospital care process, including pre-admission, ICU stay, and post-discharge.

- P01: “It was not an easy thing to have her [mother] there. I thought about, when she was here, I could look after her. But then I couldn’t.”
- P05: “Even though I couldn’t be there, I had to put my faith in the doctors and nurses to take care of my father.”
- P06: “I could barely look at him [father] in his state with all the tubes and lines in him. I just saw his face and basically there was, you know, obviously a lot of machines.”

**Essential Theme 2: Ethical Conflict Regarding Physical Restraint Use**

This theme reflected the moral and ethical challenges family members faced when their loved ones were physically restrained.

- P01: “It’s not a good thing to do that—especially if you’re old, why would you want to do that? It’s for her own good, yes, for her own good. It was a necessity. I didn’t agree that they had to do that, but they had to do it. I didn’t want that to happen to her [mother], but it came to the point that they had to do that. How else could you have a patient with an IV in the arm? If they keep pulling it out, what else can you do?”

The primary reason for physical restraint use in this study was to prevent patients from pulling at medical devices, such as intravenous lines or oxygen masks, which could compromise their treatment and safety. This longstanding practice, while clinically justified, introduced new challenges in the context of the COVID-19 pandemic.

- P02: “I’m not okay with having my husband tied up, but I don’t have any option because he needed to do that so that he can heal.”
- P04: “They deemed it necessary because she [sister] kept taking off her oxygen mask. That’s what they said. I don’t know. I wasn’t there…. If there was somebody there who would have been able to just put her mask back on, you wouldn’t have needed to put restraints on. The restraints were there so that no one—that they [nursing staff] can just go ahead and watch TV or whatever it is what that they did and go about their merry little way.”
- P06: “I don’t know if there were any other purposes for restraining. But the purpose they gave us sounded reasonable and everything. And then we didn’t really question that…. I would’ve questioned it if he was awake and everything.”

**Essential Theme 3: Barriers in Obtaining Consistent Information**

This theme highlighted the frustrations and difficulties families experienced in getting clear and consistent information from healthcare providers.

- P04: “I couldn’t get straight answers. Every time I asked about the restraints, I got different responses. It was frustrating and scary not knowing.”
- P03: “Disgruntled to send my mom to the hospital. I felt she was very sad, very despondent…. The nurse and the doctor did not do good service. The hospital could not heal the people, and one by one, they left.”
- P07: “I knew they were busy. They had schedules, and they had to get to see all the patients and everything. But a little better communication would have, you know, made the situation a little better, you know.”
- P10: “I tried to call the nurse, call the doctor. I left the message, but the doctor was not as fast to give me the response.”

**Essential Theme 4: Lack of Control and Trust in Healthcare Providers**

This theme exemplified the sense of helplessness and lack of control experienced by family members due to visitation restrictions and their inability to be with their loved ones.

- P06: “It was hard, but I had to trust that they were doing the best they could for him [father].”
- P08: “Not being able to be there to advocate for him [father], I felt like I had no control over what was happening to him.”

**Essential Theme 5: Emotional Turmoil**

This theme crystallized the intense emotional distress experienced by family members when they saw their loved ones restrained in the ICU.

- P04: “How was it done? Were they fighting with her [sister]? Because someone who does not want to be restrained is not going to want to be restrained. So, were you talking about four, five, or six men? Like, what are we talking about here? I thought about that. I don’t know how it was done.”
- P06: “I could barely look at him [father] in his state with all the tubes and lines in him. I just saw his face and basically there was, you know, obviously a lot of machines.”

**Essential Theme 6: Longing for Connection**

This theme illustrated the deep yearning participants had for closeness with their loved ones. In Table 3, many participants (7 out of 10) could only visit their loved ones virtually due to COVID-19 restrictions. This physical separation added another layer of complexity to their experiences, as they were unable to provide direct comfort and support.

- P02: “All I wanted was to hold her [wife’s] hand, to tell her it was going to be all right. But I couldn’t, and it hurt so much.”
- P07: “I just feel a lot of sadness because I didn’t know exactly how she [mother] felt or what she wanted in that moment.”

For many participants, virtual video calls became the only way to connect with and see their isolated loved ones. While the participants did not explicitly contrast the virtual versus in-person experience, the inability to physically be with their loved ones clearly exacerbated their yearning for closeness during an already-distressing time. The virtual modality, while allowing some connection, could not fully replace the profound need for direct physical presence, comfort and support that family members craved to provide their hospitalized loved ones.

**Essential Theme 7: Need for Transparency**

This theme reflected participants need for honest, clear communication from healthcare providers.

- P09: “I needed them to be honest with me, to explain why this was necessary. That would have made a difference.”
- P07: “Some days, we tried to get in touch, and we weren’t able to, and the nurses weren’t fully aware of the whole situation, so they couldn’t provide us anything. It was only up to the doctor, and doctors are always busy. But they did get back, you know, and I just wished there was a little more…urgency, I guess.”

All combined, these quotes demonstrate the families’ internal conflicts, ethical concerns, and communication barriers regarding the use of restraints. This, then, provides a solid foundation for the conclusions drawn from the study. By linking these personal testimonies to the broader themes identified, the conclusions are more robustly supported by the results.

## 4. Discussion: Interpreting Family Experiences in the ICU Context

This research aimed to understand the family experiences associated with the use of physical restraints on their loved ones in ICUs during the COVID-19 pandemic. Adopting a hermeneutic phenomenological approach was instrumental in casting light on the intricate emotional landscapes navigated by these family members.

The significant emotional burden of ICU interventions on families has been well-documented and emphasizes the need to develop effective communication strategies for sensemaking [7,8,10,11,12,13]. Weick [8] discusses enacted sensemaking in crisis situations, which is particularly relevant in the context of the COVID-19 pandemic. Davidson and Zisook [11] highlight the importance of implementing family-centered care through facilitated sensemaking. The present findings echoed the insights of McAdam et al. [12] into the profound symptom experiences of families of high-risk ICU patients, reinforcing the imperative to embed empathetic communication and active family participation in ICU care protocols, particularly during global health crises such as the COVID-19 pandemic.

In line with the principles of patient-centered care, Weick’s [7,8] sensemaking framework was instrumental in guiding the thematic analysis. The framework postulates that sensemaking is not merely about the assimilation of events but about the interplay among action, interaction, and retrospective understanding [8]. During the unprecedented COVID-19 crisis, traditional familial roles were disrupted, and the usual channels of communication and engagement were significantly obstructed, challenging the sensemaking process.

The absence of a physical presence in the ICU marked a significant deviation from traditional practices involving family participation in patient care. This lack of presence resulted in a profound feeling of isolation among family members, which was a critical aspect of the “Longing for Connection” theme. This finding is aligned with research highlighting the critical importance of familial presence for the well-being of both the patient and their relatives. Additionally, the study illuminated the heightened distress families experienced over the use of physical restraints on their loved ones—a distress that was not alleviated by the hospitals communication practices in place during pandemic constraints [13].

The necessity for physical restraints in the ICU, while clinically justifiable, was met with ambivalence by family members. This ambivalence is consistent with the literature that discusses ethical dilemmas surrounding physical restraints and associated psychological impacts on patients and their families [14]. Salehi et al. [14] further explore the factors contributing to the ethical challenges faced by critical care nurses when considering the use of physical restraints, highlighting the multifaceted nature of this issue”.

### 4.1. Impact of the Pandemic Context

During the pandemic, the high patient loads and rapidly changing protocols exacerbated communication difficulties between healthcare providers and family members. This context influenced the perceptions and experiences of the families, adding layers of complexity to the already challenging ICU environment. The absence of a physical presence in the ICU marked a significant deviation from traditional practices involving family participation in patient care. This lack of presence resulted in a profound feeling of isolation among family members, which was a critical aspect of the “Longing for Connection” theme. This finding is aligned with research highlighting the critical importance of familial presence for the well-being of both the patient and their relatives. Additionally, the study illuminated the heightened distress families experienced over the use of physical restraints on their loved ones—a distress that was not alleviated by the hospital’s communication practices in place during the pandemic constraints. In non-pandemic times, hospitals might be less busy, potentially allowing for clearer and more consistent communication between healthcare providers and family members. This speculation suggests that some of the distress experienced by family members during the pandemic could have been mitigated with better communication practices. The unique context of the pandemic played a crucial role in shaping the findings of this study, emphasizing the need for improved communication strategies in crisis situations.

### 4.2. Policy Implications

The findings advocate for a re-evaluation of current policies regarding the application of physical restraints, especially to consider the importance of addressing family needs during ICU stays. Based on the studies findings, policy changes should focus on the following:

- Strengthened communication practices: Implementing clear, compassionate, and regular communication strategies between healthcare providers and family members to alleviate psychological strain and ensure family involvement in care decisions.
- Alternate Interventions: Identifying and prioritizing alternative interventions to physical restraints, ensuring that restraints are considered only as a last resort.
- Family-centered care principles: Incorporating family-centered care principles into pandemic response planning and ICU practices to better accommodate family needs and support family members’ psychological well-being. This aligns with the recommendations by Hart et al., who emphasize the importance of family-centered care during the COVID-19 era [15].

### 4.3. Proposal for Better Communications with Family Members

A major theme that emerged from the interviews was the need for clear, compassionate, and regular communication with family members. Families expressed a desire for honest and transparent communication regarding the necessity of restraints and other critical care decisions. This can be achieved through the following:

- Regular updates: ensuring that healthcare providers implement protocols that give consistent updates to family members, including explanations of medical decisions and care plans.
- Use of technology: facilitating virtual meetings and updates through secure video-conferencing platforms to maintain family involvement, even when physical presence is not possible.
- Empathetic communication training: providing training for healthcare staff on how to communicate effectively and empathetically with family members, especially in high-stress situations. Via-Clavero et al. highlight the importance of understanding critical care nurses’ beliefs regarding physical restraint use, which can inform communication strategies with family members [16].
- Family liaison roles: establishing dedicated roles to maintain communication with families while ensuring that their questions and concerns are addressed promptly.

### 4.4. Limitations

The limitations of this study were intrinsically linked to the unique context of the ongoing COVID-19 pandemic during the data collection phase. Notably, the pandemic-imposed restrictions on hospital visits significantly impacted the experiences of the participants. The inability of most family members to be physically present in the ICU, combined with challenges in arranging virtual visits, meant that updates on the patients’ conditions were often scarce or delayed. Consequently, many family members’ perceptions and understandings of the use of physical restraints were indirectly influenced by the limited available information, rather than from direct observation or interaction with healthcare staff (refer to Table 3 for an overview of participants’ experiences with physical restraint interventions and hospital visitations).

Furthermore, the timing of the interviews may have coincided with the acute phase of grief for several participants, particularly those who had recently lost their loved ones. This overlap could have influenced their recollections and interpretations, coloring their perspectives with the intensity of their emotions. While this does not diminish the validity of their experiences, it is an important consideration when interpreting the depth and breadth of the emotional responses captured in this study.

## 5. Conclusions

This study documented the complex challenges encountered by family members of ICU patients who experienced physical restraint during the COVID-19 crisis. It delineated crucial themes that included emotional turmoil, ethical dilemmas, and obstacles in communication, all of which significantly impacted the families’ experiences. These findings underscored the need for a holistic approach to physical restraint use that carefully considers its psychological and ethical repercussions on patients and their families.

The study also highlighted the necessity for protocols that enable clear, compassionate, and regular communication between healthcare providers and family members. Nurses, often the primary point of communication, are in a pivotal position to implement strategies that alleviate psychological strain on families. This encompasses providing frequent updates on patient status, enabling connections through technology when in-person visits are not possible, and valuing family contributions in care decisions.

From a policy perspective, the findings of this study prompted a re-evaluation of current policies regarding the application of physical restraints, with a focus on identifying and prioritizing alternative interventions to ensure that restraints are considered only as a last resort. It is also recommended that policies require the active involvement of families in care discussions, honoring their desire for openness and participation in decisions affecting their loved ones.

The study provided critical insights that may inform future policy adjustments aimed at addressing family needs during ICU stays. Future research should explore strategies that could reduce reliance on physical restraints in the ICU, while concurrently analyzing the supportive role of the family to enhance the psychological welfare of patients. Additionally, the study highlighted the necessity for adaptive strategies by healthcare systems to maintain sensemaking processes and support family members’ psychological well-being. This aligned with current scholarship advocating for adaptive communication strategies in times of healthcare crisis [15]. The pandemic’s ongoing restrictions require innovative approaches to uphold a standard of care that includes family engagement and shared decision-making.

Longitudinal research could provide valuable insights into the enduring consequences of physical restraints on both patients and their families, informing enhancements in ICU care practices.

The study championed a reimagined approach to ICU care, one that embraces a holistic, family-centric model consistent with the ethos of patient-centered care. It highlighted the importance of preserving the personal element of care delivery, even amid the exigencies of a pandemic. The integration of these research findings into nursing protocols and healthcare policies is pivotal to achieving the objectives of reducing the use of physical restraints in the ICU while upholding a healthcare paradigm that is empathetic and ethically sound.

Finally, these findings underscore the importance of clear, compassionate, and regular communication between healthcare providers and family members. Such communication is essential not only during a pandemic but also in post-pandemic ICU care. Improving family engagement and transparency can help alleviate psychological distress and improve overall satisfaction with the care process. These insights could inform and enhance ICU practices in future healthcare crises as well as more routine situations so that, family needs are consistently addressed and integrated into patient care.

## Figures and Tables

**Table 1:** Demographic information of participants and their loved ones.

Participant ID	Nationality	Loved One’s Relation to Participant	Age of Loved ONE	Diagnosis	ICU Unit	Discharge Status
P01	Guyanese	Mother	90	Pulmonary Edema	IMCU	Transferred, Discharged to Home
P02	Ghanaian	Husband	60	Stroke	IMCU	Transferred to Nursing Home
P03	Cantonese	Mother	63	PNA due to COVID-19	ICU	Deceased
P04	Hispanic	Sister	57	Acute Respiratory Failure due to COVID-19	ICU	Deceased
P05	Bangladeshi	Father	68	ARDS due to COVID-19	ICU	Deceased
P06	Bangladeshi	Father	68	ARDS due to COVID-19	ICU	Deceased
P07	Black/Biracial	Mother	65	PNA	ICU	Deceased
P08	Bangladeshi	Father	82	STEMI ST Elevation/Myocardial Infarction	CCU	Discharged to Home
P09	Togolese/West African/Black	Mother	81	AV Block 2nd Degree	MICU	Recovered, Transferred to Step-down Unit
P10	Guyanese/ West Indian	Mother	69	Acute Respiratory Failure due to COVID-19	IMCU	Deceased

**Table 2:** Thematic analysis of family member experiences in ICU physical restraint scenarios.

Theme Category	Themes	Essential Themes
Hospitalization Journey	Navigating the patient’s ICU stay and post-discharge period	Managing complex hospital care challenges throughout the continuum of hospital care
Ethical Dilemmas	Restraint necessity vs. moral concerns	Ethical conflict regarding physical restraint use
Communication Issues	Frustration with healthcare communication	Barriers in obtaining consistent information
Perceived Control	Grappling with powerlessness during visitation restrictions	Lack of control and trust in healthcare providers
Emotional Responses	Shock, helplessness, and anxiety	Emotional turmoil
Connection and Support	Desire for physical and emotional closeness	Longing for connection
Need for Transparency	Desire for honest communication	Need for transparency in healthcare decisions

**Table 3:** Participants’ experiences with physical restraint intervention.

Participant ID	Reason for Restraint	Visit Type
P01	Pulling at medical devices	Physical and Virtual
P02	Pulling at medical devices	Virtual
P03	Pulling at medical devices	Virtual
P04	Pulling at medical devices	Virtual
P05	Pulling at medical devices	Virtual
P06	Pulling at medical devices	Virtual
P07	Pulling at medical devices, trying to get out of bed	Virtual
P08	Pulling at medical devices	Virtual
P09	Pulling at medical devices	Physical
P10	Pulling at medical devices	Physical and Virtual

## Data Availability

The data presented in this study are available on request from the corresponding author. The data are not publicly available due to privacy and ethical restrictions.
